# Challenges and Legal Gaps of Genetic Profiling in the Era of Big Data

**DOI:** 10.3389/fdata.2019.00040

**Published:** 2019-11-12

**Authors:** Murat Sariyar, Irene Schlünder

**Affiliations:** ^1^Institute of Medical Informatics, Bern University of Applied Sciences, Bienne, Switzerland; ^2^TMF – Technologie- und Methodenplattform e.V., Berlin, Germany; ^3^BBMRI-ERIC, Graz, Austria

**Keywords:** big data, data protection, data privacy, genetic profiling, anti-discrimination laws

## Abstract

Profiling of individuals based on inborn, acquired, and assigned characteristics is central for decision making in health care. In the era of omics and big smart data, it becomes urgent to differentiate between different data governance affordances for different profiling activities. Typically, diagnostic profiling is in the focus of researchers and physicians, and other types are regarded as undesired side-effects; for example, in the connection of health care insurance risk calculations. Profiling in a legal sense is addressed, for example, by the EU data protection law. It is defined in the General Data Protection Regulation as automated decision making. This term does not correspond fully with profiling in biomedical research and healthcare, and the impact on privacy has hardly ever been examined. But profiling is also an issue concerning the fundamental right of non-discrimination, whenever profiles are used in a way that has a discriminatory effect on individuals. Here, we will focus on genetic profiling, define related notions as legal and subject-matter definitions frequently differ, and discuss the ethical and legal challenges.

## Introduction

**Figure 1:**
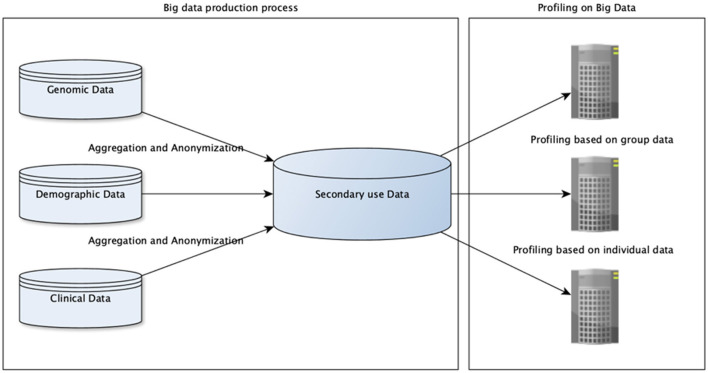
Two different domains of data processing are depicted. Processes on the left for generating secondary use data out of different sources (only three general ones are given here) are related to data protection regulations. Processes on the right for inferring profiles are related to anti-discrimination regulations.

In healthcare, potentials of big data receive more and more attention as the volume, variety, and velocity of healthcare data is increasing steadily. Big companies, for example Google, Apple and IBM are investing huge resources in this area in order to exploit their data assets. As data have an intrinsic value, for example, as resource of gaining knowledge about human needs and preferences, buying and selling them or using them for various purposes is tempting. Customizing product offers and pricing based on our habits, needs, and characteristics usually increases profits in various different areas, but might entail detrimental effects for the customer and unacceptable implications, such as ethnical and gender discrimination, for the society as a whole. This danger does not primarily stem from the central risk data protection law is addressing: the risk of unwarranted disclosure of person-related data. It is rather the risk of creating general profiles, which are later used in decision making, and might lead to unfair and discriminatory treatment. For such profile creation, re-identification is not needed, i.e., in most cases, anonymized and aggregated data are sufficient. An overall picture is given in Figure 1. Therefore, privacy preserving techniques are rather toothless to mitigate these risks—one central purpose in generating anonymized data is to allow inference of new patterns. In this paper, we deal with the question of how this kind of risks associated with (genetic) profiles can be mitigated while harnessing their advantages for health research and healthcare.

“By definition, big data in healthcare refers to electronic health data sets so large and complex that they are difficult (or impossible) to manage with traditional software and/or hardware…” (Rubin and Desser, 2008). One the one hand, many promises related to these large and complex data still need to be realized; on the other hand, there are fears regarding the increased misuse of those data. Both aspects are consequences of the complexity that renders it impossible to assess upfront the realizable benefits and misuses. Hence, discussing big data challenges has a high risk of projecting prejudices and it is important to reduce this risk by presenting and exploring the relevant notions and legal frameworks.

Profiling in general is “as old as life” (Gutwirth and Hildebrandt, 2010); it is the deduction of information based on some characteristics of an individual or a group of individuals, which are either known beforehand or also deduced. Generally, profiling may be based on different grounds: human experience, cultural stereotypes or statistical correlations. The result is always a certain conclusion with respect to an individual based on the assumption that the individual can be classified as belonging to a certain group of individuals, who are assumed to exhibit the same characteristics with a certain probability (European Commission, 2018a). The possibilities introduced by big data and methods such as data mining and deep learning allows new levels of insights that shape our view of the present and the future. Thus, the impact of profiling on society has been enlarged in the era of big data and data mining methods. There are many different contexts for profiling, e.g., information science, healthcare settings, forensic science, marketing, etc. Big data increases the usage possibilities of profiling for detecting new patterns and automated decision making (Hildebrandt, 2008).

Profiling in healthcare is highly related to personalized medicine, allowing better tailored decisions instead of those based on average characteristics. Potential applications are: biomarker-guided diagnosis decision and therapy selection, prognostication, resistance detection, disease monitoring, risk assessment, recurrence detection, and early detection (Wjst, 2010). Main challenges of profiling based on big data stem from the deduction of knowledge leading to certain decisions that are sensitive in an ethical and legal sense: it can lead to non-transparent decision making and even unfair treatment and discrimination of individuals. “Profiling can perpetuate existing stereotypes and social segregation. It can also lock a person into a specific category and restrict them to their suggested preferences. This can undermine their freedom to choose, for example, certain products or services such as books, music or newsfeeds. It can lead to inaccurate predictions, denial of services and goods, and unjustified discrimination in some cases” (European Commission, 2018a).

It is well-known that many cultural assumptions are statistically invalid and rather based on ideology and therefore discriminating. Discrimination, however, can also occur, when conclusions are based on valid correlations. This is the case, if such correlations are in conflict with basic ethical decisions of our society. Inequality based on gender for example is considered unethical or even unlawful, even though there might be a statistical difference in certain areas. Algorithms applied to big data sources such as data mining techniques aggravate the problems of profiling because new forms of knowledge deduction become possible that allow faster and automated ways of decision making with a lack of transparency, increasing the risk of unwarranted disclosure, and unfair use of sensitive information. One example of an unfair discrimination is the decision to charge citizens having an increased risk of developing diabetes due to their genetic disposition with a higher insurance rate. For example, in the US, the Affordable Care Act of 2010 (ACA) does not prevent insurers from charging more for such expected conditions (Saloner and Daniels, 2011). This question cannot be answered by statistical evidences only but must also refer to ethical values and legal frameworks. Transparency in this case means that such discriminatory practices and the methods behind them should be disclosed to the public.

One way to consolidate such questions of discrimination and transparency of profiling is to look into the decision-making process. Rational decisions are a result of opinions (including ethical values), experiences and empirical evidences. They are cognitive processes of identifying and choosing a belief or a course of action among several alternatives. By shifting toward empirical facts and analyses, it becomes urgent to reconsider the way the other components of decisions should be involved. Statistical results seem to show an inherent credibility and correctness due to the mathematical methods by which they are developed. People tend to accept profiles and decisions based on such results without seeking explanation or reasons for the conclusions based on them. Providing a reasoning for certain decisions beyond “facts” (which are themselves a construction), however, is a basic requirement for preventing unfair discrimination and allowing actionable transparency. It is therefore necessary to balance data-driven results with norms and thus keep control of their use in decision making. To this end, we suggest new approaches for addressing the threats of genetic profiling and also show the shortcomings of the current European legal framework, mainly GDPR (General Data Protection Regulation).

## What Is Genetic Profiling?

The term genetic profile is generally used to denote genetic signatures or information as a combination of genetic characteristics related to a human being. It is important to emphasize that profiling is the process by which such a combination of characteristics is associated with target attributes used for decision making, e.g., concerning the risk of developing a certain disease. The notion *profiling* can be summarized by following two descriptions (European Commission, 2018a):

“…a procedure which may involve a series of statistical deductions. It is often used to make predictions about people, using data sources to infer something about an individual, based on the qualities of others who appear statistically similar”

“…a means gathering information about an individual (or group of individuals) and analyzing their characteristics or behavior patterns in order to place them into a certain category or group, and/or make predictions or assessment about, for example, their ability to perform a task, interests, likely behavior.”

In health care, physicians use profiling as a part of their professional duties. Another term that can be used is “risk stratification,” i.e., using certain patient characteristics for classifying different types of disease forming (Braithwaite et al., 2016). Naturally, group-related characteristics often lead to the risk of discrimination as soon as decisions with a direct impact on the respective individuals are taken.

Genetic profiling is used in healthcare and biomedical research for associating genetic characteristics with increased or decreased likelihood of developing and overcoming certain diseases. Rarely, diseases are monogenic, i.e., the diseases are attributable to genetic variants at one locus with large effects on disease status. Cystic fibrosis is an example of such a monogenic disease. In the majority of cases, genetic disorders are polygenic, meaning they are likely associated with the effects of multiple genes in combination with lifestyles and environmental factors. While there are many forms of DNA-related markers that are relevant for certain phenotypes and identification of individuals, generally only a tiny fraction of DNA is relevant for health care and research, e.g., Genes, SNPs, Short Tandem Repeats (STR), and whole genome sequences. These markers are directly related to the DNA without any translation step in-between (in contrast to, for example, RNA variants), which allows to focus on stable characteristics at the molecular level. STRs are highly relevant, because they are frequently used for genetic fingerprinting of individuals (Sariyar et al., 2017).

With big data and related algorithms, especially stemming from the domain of artificial intelligence (AI), genetic profiling can and in some cases must be achieved by automated decision making. Using methods like deep neural network are additionally related with a black-box character, making it difficult to achieve transparency regarding the mechanism for risk prediction. We will discuss implications of these difficulties in the next section.

For making the discussion of potentials and problems of genetic profiling for individual as well as social profiling more concrete, we will use diabetes mellitus as a disease for which diagnosis and treatment can be tailored with the help of genetic profiles. “Diabetes mellitus is a chronic disease of major global health concern due to its increasing prevalence in both developing and developed countries, with a projection increase of 214% from the year 2000 to 2030” (Leung, 2016).

### Use Case for Individual Genetic Profiling for Diabetes Mellitus

In contrast to single-gene profiles for Mendelian disorders with relatively certain prediction of developing a disease, genetic profiling for diabetes mellitus type 2 (DMT2) only allow to generate disease risk information (Haga, 2009). Among the best studied causes are two closely linked single nucleotide polymorphisms (SNPs) in the transcription factor 7-like 2 (TCF7L2) gene (O'Rahilly et al., 2005). Over 20 studies have corroborated the association between these two SNPs in TCF7L2 and increased DMT2 risk. Several questions arise here, e.g.:

- How and when should predispositional screening for disease prediction be used?
- To whom and how should the risk information be communicated?

### Use Case for Social Genetic Profiling for Diabetes Mellitus

Here, social profiling does not refer to the construction of user's profile using social data, but to the process of attributing genetic characteristics leading to phenotypes otherwise not directly attributable to a social group (Leung, 2016). With respect to diabetes mellitus, the aboriginal population of Canada (including the First Nations, Inuit and Metis) represents a frequently investigated example. Among the Aboriginal population of Canada, diabetes mellitus contributes significantly to their higher morbidity when compared to the non-Aboriginal Canadians. One explanation is the *thrifty gene* theory proposed by Neel (1962): Aboriginal people had maintained a hunter-gatherer lifestyle with no guarantee of the constant food supply, and hence via evolution they acquire a thrifty gene to conserve energy through periods of starvation and environmental hardships. In view of this, Hegele et al. identified a gene mutation (G319S) in the hepatic nuclear factor-1 alpha which translates to decreased insulin sensitivity (Hegele et al., 1999, 2003). Overall, the crude prevalence for diabetes ranges from 2.7 to 19%, which is 3–5 times higher than non-Aboriginal cohorts (Oster et al., 2012). In order to reduce this high prevalence, the Federal Government of Canada had launched the Aboriginal Diabetes Initiative (ADI) in 1999 as part of the bigger Canadian Diabetes Strategy to provide a better framework for surveillance, public education, and community-based management of diabetes. Following questions arise:

- How to prevent that this public knowledge is used for the purpose of discrimination?
- Generally speaking, should it be allowed to have racial screening?

## Legal Approaches and Gaps for Addressing Genetic Profiling

### Data Protection Law

Profiling is a well-known concept in data protection law closely linked to the application of statistical means and algorithms and has already been addressed in the EU Data Protection Directive of 1998. But the abundance of available information in the world wide web and other sources together with an ever-increasing knowledge of how to use them has been shedding new light on it in the past years. EU Data protection law, however, has not been able to keep pace with this shift. The same seems to be true for other jurisdictions. The GDPR sticks to the concept of controlling the ways personal data is processed, mainly by the requirement of consent, if no other legal allowance is provided. In the era of big data, insisting on the concept of personal data that is protected and distinguished from anonymous data, which is free to be used, is not appropriate to protect citizens against the rule of algorithms and the wrong use of their results.

It is Art. 22 of the GDPR that addresses profiling by prohibiting decisions “solely based on automated decisions,” not covering other decision-making contexts using profiles. “As soon as a human being reviews and takes account of other factors in making the final decision, that decision would not be based solely on automated processing” (European Commission, 2018a). For these non-automated decisions, the creation and usage of profiles remains untouched by Art. 22. In addition to that, the application of data protection law is limited to the processing of personal data. However, the use of personal data is often not needed for profiling; anonymized data are often good enough to create profiles.

As a result, the GDPR provides only limited means to prevent undesired usage of profiles as well as the creation of profiles through the use of non-personal, i.e., anonymous, data. At least, the principle of transparency could support individuals in questioning decisions based on profiles. The principle of transparency, as it is mentioned in Art. 5, is one of the basic rules in the GDPR. Under Articles 15 (Rubin and Desser, 2008) h, and 14 (Gutwirth and Hildebrandt, 2010) g, data controllers must provide “meaningful information about the logic involved, as well as the significance and the envisaged consequences of such processing” to the data subject. But neither of these provisions seems to contain the right to get an explanation for a particular decision that has been made. The only suggestion of such a right appears in Recital 71 of the GDPR, which says that appropriate safeguards should include the ability of data subjects “to obtain an explanation of the decision reached after such assessment” in cases of fully automated decisions. Other forms of decisions are again not addressed. The principle of transparency under the GDPR has only a limited scope as the Art. 29 WP has described: “…the controller must always be able to demonstrate that personal data are processed in a transparent manner in relation to the data subject. Connected to this, the accountability principle requires transparency of processing operations in order that data controllers are able to demonstrate compliance with their obligations under the GDPR” (European Commission, 2018b). There is no general requirement of letting individuals know, what presumptions have led to a certain decision, e.g., having full transparency how insurance rates are calculated seems to be no right under the GDPR. In addition, there are a number of legitimate reasons to hide a decision-making process, for example intellectual property rights regarding methods and algorithms used.

In view of this, concerns of citizens that their characteristics, i.e., belonging to an ethnic group with an assumed genetic risk, might be used without letting them know how and to what extent, seems to be justified. The protection against profiling is too weak and limited in scope to be effective in controlling whether data are being misused. Even though, the creation of a personal genetic profile based on personal data of the concerned individual in an automated way might require the consent of this individual according to Art. 22 GDPR, the creation of genetic group profiles is not covered by the GDPR. Such group profiles might be a factor in various decision-making processes, without letting the customers know. It then depends on the effectiveness of keeping one's own genetic characteristics secret whether they influence insurance rates. In many cases, such as ethnic or family membership, this is unrealistic. In addition, there is even a general obligation for customers to reveal any known health impairments while seeking insurance coverage. This is to a certain extent justified, since otherwise a patient would be empowered to conclude for example a life insurance contract knowing that he/she is going to die soon. The question is, whether or when a genetic profile revealing certain risks must be considered detrimental to the health status.

### Anti-discrimination Law

Many jurisdictions contain a basic provision for prohibiting discrimination, as it is laid down in various human rights catalogs, e.g., Article 14 of the European Convention on Human Rights. It is a basic principle of a democratic and open society that people should exclusively be judged and treated according to their free behavior—and not by inherited or otherwise unchangeable attributes such as gender or race or by their religious or political convictions. Article 9 of the GDPR reflects the particular discriminatory potential of such data by providing an enhanced protection of special categories of personal data, including health data and genetic data. But here again, the scope of the protection in the GDPR is very limited, as the GDPR does not address the discrimination on the basis of such data as such.

The fundamental right to non-discrimination does not explicitly comprise genetic information. Gender, race, and ethnic origin are the central characteristics addressed by discrimination laws. By using genetic information, these visible features can often be deduced quite well. Hence, genetic data are an example of proxy-data that can be used instead of visible phenotypes without directly exhibiting the discriminatory purpose, as they are frequently only available after sequencing within an unsuspicious scientific context.

Since the discriminatory potential of genetic profiles, especially in the field of insurance contracts and employments, is quite obvious, there are attempts to restrict the use of such profiles across jurisdictions. In 2008, the Genetic Information Nondiscrimination Act (GINA) was adopted in the USA to prohibit discrimination by health insurers or employers. Health insurers (group, individual, and Medicare issuers) are prohibited from adjusting premiums or contribution amounts, requesting or requiring an individual or a family member of an individual to undergo a genetic test, obtaining and using genetic test results in making a determination regarding payment, or requesting, requiring, or purchasing genetic information for underwriting purposes. The statute does not cover schools, mortgage lending, or housing. And it excludes other forms of insurance like life insurance, long-term care, and disability insurance. Therefore, the American Medical Association has pointed to the shortcomings at an early stage (Prince and Roche, 2014). In Germany, the “Gendiagnostikgesetz” adopted in 2009, contains similar clauses including life insurance. In the UK, The Concordat and Moratorium on Genetics and Insurance 2014 (Genetics and Insurance ABI, 2018) regulates how genetic information are to be used for calculating risks. These Anti-discrimination acts or declarations, however, are as limited in scope as the GDPR is. They do not cover the use of genetic profiles in all cases, but only to the extent explicitly regulated.

In addition, antidiscrimination law has often been considered toothless, since it is only applied when it can be proved that a decision has been based on discriminatory presumptions. This proof is always difficult. It might be even more difficult if the decision-making procedure is not transparent.

### Implications of the Use Case for Individual Profiling

One central issue for individual profiling is related to the reason of generating a profile and secondary use scenarios for profiles. From an ethical point of view, informed consent of the individual or its guardian is crucial. It should be known for what primary purpose and with which benefits as well as disadvantages such a profiling is associated. In the research setting, the purpose of gaining insights into average responses is more and more replaced by the goals of individualized medicine. This renders genetic profiles more important than they were before, which is one reason why the GDPR has explicitly included genetic data into the definition of sensitive data. While research as such poses rather few risks of discrimination, translating the results into the health care increases the likelihood of being discriminated, mainly because risk probabilities have to be translated into binary decisions with economic impacts. Hence, if diabetes mellitus is diagnosed with a certain probability, it depends on the context and the decision-making procedures whether and how this probability is sufficient for causing certain actions.

A health care provider can in most cases only relate to the medical implications. However, other stakeholders such as employers or insurance companies might use genetic profiles and associated risks for other forms of decisions. It depends on the legal context whether an individual have to disclose his genetic profile and the associated risk to these other stakeholders or if he can keep this information secret. Even if the legislator prohibits the usage of certain profiles in health care settings, it will rather be difficult to prevent discriminations if there are scientifically known or knowable differences in risks for different profiles. With respect to life insurance companies, there is often a gray area. Legislation such as GINA do not cover them fully, which means that there is no prohibition for requiring existing profiles in order to compute overall survival probabilities. If there are no such profiles, it would be rather unlikely that they have to be provided because of the huge number of genetic tests that could be done. This means that increased knowledge about oneself increases the possibility of discrimination. Making the individual aware of this fact and increasing his decision competencies are goals that could accompanied by legal rules for advisory committees and increased transparency requirements.

### Implications of the Use Case for Social Profiling

Due to the potential of racial discrimination, group profiling based on genetic data is one of the most prominent form of profiling in the context of health research. Knowing from research that prevalence for diabetes is 3–5 times higher for the Aboriginal population than for the non-Aboriginal population, it is difficult to prevent discrimination on that basis. Prohibitions can bring about a certain amount of certainty, but with the potentials of big data and artificial intelligence, research and data processing achieve a new level of knowledge generation. In view of this, genetic repositories as the ones of the Wellcome trust restricted access to their data (Zerhouni and Nabel, 2008). Mostly, privacy concerns are given as reasons for these restrictions. However, group profiling can still be done with anonymized and aggregation data. Discrimination risks are much harder to assess than privacy breaching risks, as there are many routes for achieving results on groups. Here again, the legislator should increase transparency requirements and it seems that this is the best he can do, as it is infeasible to list and regulate all applications that might be harmful.

## Discussion and Conclusion

There has always been a debate whether or under what circumstances research, which lead almost always to some sort of profiles, might be harmful. As the diabetes use case shows, group profiling within health research and healthcare has many positive effects, e.g., better prevention, public education and community-based management of diabetes. Hence, ethnical/genetic screening can in principle be beneficial for the citizens' well-being. Furthermore, we assume that there is no evil scientific research as such, but its results can of course always be used for malicious purposes and thus raise ethical concerns. In some rare cases, concrete research projects might pursue ethically questionable goals or might be based on questionable methods. But those seem to be rare. Research Ethics Committees or Review Boards are in charge of this. Most societies have a well-established system of research ethics, and there is an even older tradition in medical ethics.

Research is as necessary for societies as personal freedom, and research is as protected by fundamental rights as privacy is. But citizens have to be protected against an uncontrollable and unfair use of knowledge that has been derived from “their” available data, even if this data is not identifying. Otherwise, we risk ending up in a society where citizens are afraid of revealing information about their life and health status due to the concern that this information might lead to unfair treatment. This is detrimental for research as well as for any democratic society. But how could such a protection look like without leading to an overcontrolled and restricted use of information preventing free research and gaining insights beyond a highly regulated and maybe overcontrolled institutional research? How to balance the free flow of information for the sake of freedom and progress with the legitimate interests of individuals not to be treated in the basis of presumptions, they cannot even challenge due to their complexity or due to protected IP?

The simple solution is to keep research and health care data secret, e.g., by storing patient-related data at safe storage places, and preventing their use for any other purpose than for health care and research. This is exactly the approach of the GDPR: personal data collected for specific purposes such as health care or a certain research project must not be used for any other purpose. But we all know the shortcomings of this approach: non-personal data are not covered, and even genomic data are frequently considered anonymous data under various jurisdictions despite the high risk of re-identification once those data have left any protected privacy preserving system. On the other hand, the restrictions for research are often quite harsh in relation to the success of keeping crucial data secret. For example, as soon as the patient receives relevant health information derived from genetic analysis, he/she might even be obliged to reveal those risks while concluding an insurance contract, while usage of the same data in another research project might be prohibited.

Big data and related methods foster new opportunities in gaining new knowledge in secondary data use contexts that even might not be considered scientific, which means that the boundary between science and application blurs. It is difficult and often impossible to expand principles and rules for clinical research to all of these secondary use contexts; hence, the discrimination potentials are growing in an ever-increasing rate. This development makes the core question more acute: How to mitigate risks associated with (genetic) profiles while harnessing their advantages for health research and healthcare?

If society does not want to slow down developments in data science, solutions cannot rely on prohibitions of all kind applications, but has to focus on increased transparency as well as on ethical debates regarding good and bad purposes of profiling independently of economic impacts.

For answering the question of how to prevent the use of personal genetic profiles derived from personal health records or research data bases for discrimination, it has first to be decided where, by whom, by which security technique, under which format and under which rules such profiles are stored. In order to guarantee as much freedom as possible, disclosing of information should mainly dependent on the free will of the data subject. However, health insurances, life insurances, etc., want to be able to customize their offers according to the information they can receive from their customers. If such information is available, it is difficult to prevent its usage. Most important, however, is the fact, that in many cases, such as ethnic or family membership, it is unrealistic to hide certain genetic characteristics. As the diabetes case clearly shows, it is not the personal genetic profile of a certain individual that is harmful for an individual's life opportunities, but the mere fact that he/she obviously belongs to a certain group. The “typical” genetic profile due to ethnic origin is sufficient to trigger assumptions about the health status. The same will become true for an increasing number of genetic group profiles connected to obvious characteristics. In contrast to other profiles based, for example, on the outer appearance, such genetic profiles are related to a broad range of aspects, which are not immediately visible.

As AI algorithms can lead to socially unintended discrimination, a public debate on the transparency of the decision-making process and results of machine learning algorithms is necessary. It should be clear: Algorithms do not rule us but provide the result we need for decisions. The public debate has to determine how transparency with respect to the algorithms, underlying assumptions, and their results can be achieved. What we need, is a general right to get to know and assess the presumptions behind decisions affecting individuals. This is highly relevant, as it is possible to hide characteristics such as race or genetic disposition behind less suspect items that are highly correlated with them. The latter is all the more true regarding social profiling, which does not need personal data and can therefore undermine privacy of individuals that have kept their profiles secret.

In conclusion, decisions concerning individuals should always be challengeable, especially when sensitive genetic information is involved. Regulations regarding genetic profiles cannot cover all possible uses and misuses, which means that the legal gap cannot be closed only by more detailed prohibitions. In addition to that, mechanisms of oversight should be established, allowing standardized and transparent procedures for deciding whether the use of genetic profiles is based on unjustified or discriminatory assumptions.

## Author Contributions

MS and IS designed and structured the manuscript. MS and IS equally contributed to the section Introduction. MS wrote most of the section What is Genetic Profiling? IS wrote most of the section Legal Approaches and Gaps for Addressing Genetic Profiling. MS and IS equally contributed to the section Discussion and Conclusion.

### Conflict of Interest

The authors declare that the research was conducted in the absence of any commercial or financial relationships that could be construed as a potential conflict of interest.
